# The COVID-19 Assessment for Survival at Admission (CASA) Index: A 12 Months Observational Study

**DOI:** 10.3389/fmed.2021.719976

**Published:** 2021-09-30

**Authors:** Gianluca Bagnato, Daniela La Rosa, Carmelo Ioppolo, Alberta De Gaetano, Marianna Chiappalone, Natalia Zirilli, Valeria Viapiana, Maria Concetta Tringali, Simona Tomeo, Caterina Oriana Aragona, Francesca Napoli, Sara Lillo, Natasha Irrera, William Neal Roberts, Egidio Imbalzano, Antonio Micari, Elvira Ventura Spagnolo, Giovanni Squadrito, Sebastiano Gangemi, Antonio Giovanni Versace

**Affiliations:** ^1^Department of Clinical and Experimental Medicine, University of Messina, Messina, Italy; ^2^BIOMORF Department, University of Messina, Messina, Italy; ^3^Department of Medicine, University of Kentucky, Lexington, KY, United States; ^4^Department for Health Promotion and Mother-Child Care, University of Palermo, Palermo, Italy

**Keywords:** COVID-19, outcome research, mortality risk, stratification index, systemic score

## Abstract

**Objective:** Coronavirus disease 2019 (COVID-19) is a disease with a high rate of progression to critical illness. However, the stratification of patients at risk of mortality is not well defined. In this study, we aimed to define a mortality risk index to allocate patients to the appropriate intensity of care.

**Methods:** This is a 12 months observational longitudinal study designed to develop and validate a pragmatic mortality risk score to stratify COVID-19 patients aged ≥18 years and admitted to hospital between March 2020 and March 2021. Main outcome was in-hospital mortality.

**Results:** 244 patients were included in the study (mortality rate 29.9%). The Covid-19 Assessment for Survival at Admission (CASA) index included seven variables readily available at admission: respiratory rate, troponin, albumin, CKD-EPI, white blood cell count, D-dimer, Pa02/Fi02. The CASA index showed high discrimination for mortality with an AUC of 0.91 (sensitivity 98.6%; specificity 69%) and a better performance compared to SOFA (AUC = 0.76), age (AUC = 0.76) and 4C mortality (AUC = 0.82). The cut-off identified (11.994) for CASA index showed a negative predictive value of 99.16% and a positive predictive value of 57.58%.

**Conclusions:** A quick and readily available index has been identified to help clinicians stratify COVID-19 patients according to the appropriate intensity of care and minimize hospital admission to patients at high risk of mortality.

## Introduction

Since the outbreak in Wuhan city, China on December 2019 of a viral pneumonia with an unidentified etiology (1), a novel strain of coronavirus was isolated and defined Severe Acute Respiratory Syndrome Coronavirus-2 (SARS-CoV-2) by the Coronaviridae Study Group (CSG) of the International Committee on Taxonomy of Viruses (2). COVID-19 became the name of the disease that is induced by the aforementioned virus, which has caused a global pandemic accounting, as of May 8th 2021, for more than 3.2 million deaths worldwide as reported by WHO.

After more than one year from the original Chinese outbreak, national health systems are still facing devasting consequences due to the poor ability to control infection spreading and slow adaptation to the high number of hospital admissions. A lack of ability to easily stratify the patients on the first day of admission has further hampered efforts to influence the rate of mortality. Currently the overwhelming number of Sars-Cov-2 infected patients requiring hospitalization outpaces the availability of human and medical resources in several countries. All these conditions argue for the definition of a very high performing, predictive index of mortality to allow the appropriate allocation of COVID-19 resources (3). For maximum utility such an index would be constructed from elements of clinical and laboratory features available on the first day of hospitalization and applicable as a guide to reorganize dedicated COVID-19 divisions according to disease severity at admission and mortality risk (4). Paradoxically, identifying patients occupying the opposite end of the disease severity spectrum – those with very low or perhaps even no risk – can be equally useful to resource allocation. Clinicians and health care systems urgently warrant reliable scores to identify those patients with COVID-19 at highest risk for death requiring admission, to stratify them in the appropriate level of care and apply a rational optimization of resources. In addition, these instruments can also help clinician adopt quick and reliable clinical decisions and anticipate the prognosis to patients and their families.

Despite in the early phase of pandemic several other COVID-19 and non-COVID-19 specific scores have been employed in an attempt to stratify according to mortality or ICU admission risk, validation studies failed to confirm their applicability in larger populations (5–15). According to a recent review article analyzing the existing scores predicting COVID-19 mortality, the 4C mortality score, designed specifically for COVID-19, is likely to have the best performance, though it has intrinsic limitations and a relatively low positive predictive value (16–18).

Our aim was, then, to identify a COVID-19 specific score to predict mortality with improved positive predictive value and comparable very high negative predictive value, thus reducing the number of COVID-19 patients requiring medium-high intensity of care and concentrating patients at risk of mortality in adequately skilled and equipped divisions. Early stratification of patients who are either highly prone to develop critical disease, or are substantially protected from it, is of undoubted importance in guiding the clinician in delivering proper care and optimizing use of limited health resources.

Thus, we aimed to build a mortality risk prediction score for an ascertainment set of consecutive patients with COVID-19 and radiologic evidence of pneumonia based entirely upon data available on the day of admission.

## Materials and Methods

This is a single center, longitudinal, observational study involving consecutive admitted patients aged ≥18 years and diagnosed with Sars-Cov-2 infection and radiologic evidence of pulmonary involvement. The study enrollment period was 1 year time from March 2020 to March 2021 in the critical medicine division of the University of Messina. Patients directly admitted in ICU were not included in our study. In addition, we excluded patients who were either admitted with non-COVID-19 symptoms and had incidental asymptomatic diagnosis (e.g. hip fracture without clinical features of COVID-19), those admitted for isolation/control reasons, and also those with COVID-19 who were discharged from either ambulatory care or the emergency room, without admission. Among 249 eligible patients, 5 were excluded due to missing data at admission. A total of 244 patients with confirmed Sars-Cov-2 infection and radiologic evidence of pulmonary involvement were thus enrolled in the study. Oropharyngeal and nasal swab samples were taken from all patients upon hospital admission. Patients with a positive result of swabs, confirmed by real time reverse transcriptase polymerase chain reaction, and radiologic evidence of pneumonia were included in the study. The study was approved by the institutional review board of the University of Messina and informed consent was obtained from all subjects. They all agreed to routine access to their record and analysis of their medical history data at the time of admission.

### Data Collection

The demographic (age, sex, home isolation, in-hospital stay, smoking exposure, body mass index and comorbidity), clinical (vital parameters and signs/symptoms), laboratory parameters at admission, Ordinal Scale for Clinical Improvement (19), therapeutic regimen and the final outcomes (the deceased/survivors, ICU transfer) of the enrolled patients were prospectively collected. The following new onset signs/symptoms were included: fever, cough, shortness of breath, confusional state and Coma Glasgow Scale, diarrhea, fatigue, headache, nausea or vomiting, sore throat, sputum production, arthralgia or myalgia. Comorbidities collected were: chronic cardiac disease, chronic respiratory disease, chronic renal disease, dementia, chronic neurological conditions, connective tissue disease, thyroid disease, diabetes mellitus and malignancy.

The laboratory parameters included were: hemoglobin, platelet count, red cell count, white cell count, lymphocyte count, hematocrit (HCT), C-reactive protein (CRP), procalcitonin, aspartate aminotransferase (AST), alanine aminotransferase (ALT), lactate dehydrogenase (LDH), albumin, ferritin, fibrinogen, urea, creatinine, Chronic Kidney Disease Epidemiology Collaboration (CKD-EPI) equation (CKD-epi) for the assessment of Estimated glomerular filtration rate troponin, NT-Pro-BNP and D-dimer, PaO2/FiO2 ratio. The clinical information used to calculate prognostic scores was taken from the day of hospital admission.

### Outcomes

The primary outcome of the study was in-hospital mortality. This outcome was selected because of its face validity. We considered of primary interest the stratification of patients according to the mortality risk in order to be able to escalate intensity of care and therapeutic regimen for patients likely to develop severe illness from SARS-CoV-2 infection.

### Independent Predictor Variables

A reduced set of potential predictor variables was selected *a priori*, including patient demographic information, common clinical investigations, and parameters consistently identified as clinically important in COVID-19 cohorts, as previously reported (20).

Candidate predictor variables were selected based on two common criteria: variables known to influence outcome in pneumonia and flulike illness; clinical biomarkers previously identified within the literature as potential predictors in patients with COVID-19 (21–23).

### Statistical Analysis and Model Development

Continuous and categorical variables were presented as median (IQR) and n (%), respectively. We used the Mann-Whitney U test or Fisher's exact test to compare differences between survivors and non-survivors where appropriate.

The primary intention was to create a pragmatic model for use during admission to stratify patients for intensity of care, maximizing for sensitivity and specificity in order to exclude those not requiring a medium-high intensity of care. We used the following steps to build the model: firstly, univariable logistic regression analysis was performed for all the variables that were significant in the between-group analysis. Since age was independently associated with the outcome of interest (the deceased vs. survivors), we performed age-adjusted univariable logistic regression for the variables significant in the first round of univariable logistic regression analysis after categorizing them according to laboratory reference range or clinical criteria where appropriate.

To enhance a pragmatic approach, the significant age-adjusted variables were plotted in their categorized form, according to the reference range of the test (present/absent), in multivariable logistic regression analysis with backward stepwise method.

The model retained 7 variables, thus minimizing the risk of overfitting according to the number of events occurred (*n* = 73). A score was then created by multiplying the odds ratio of each relevant variable. Receiver operating characteristics (ROC) curves were used to assess the accuracy of model predictions and area under curve (AUC) with negative predictive value (NPV), positive predictive value (PPV), specificity and sensitivity was given and compared to the results of age, SOFA and 4C mortality scores according to De Long et al. (24).

## Results

Demographics characteristics, vital parameters, clinical features and comorbidities at admission for the entire study population and for the group of deceased patients (73) and survivors (*n* = 171) are collected in Table 1, while Table 2 shows the laboratory results. The mortality rate of our study population was 29.9%, while 13.5% were transferred in ICU. Patients were treated with the following medications: 87% with corticosteroids, 85% with heparin, 16% with hydroxychloroquine. No patients were treated with remdesivir, tocilizumab, plasma hyperimmune or monoclonal antibodies.

**Table 1:** Demographic and clinical features of COVID-19 patients at admission.

	**Total (*n* = 244)**	**Deceased (*n* = 73)**	**Survivors (*n* = 171)**	***p***
**Demographics**				
Age, median (IQR)	72.3 (24.9)	79.7 (12.7)	69 (23.08)	< 0.001
Over 65 years, n (%)	155 (67.6)	66 (90.4)	99 (57.9)	< 0.001
BMI, median (IQR)	25.4 (4.6)	25.7 (5.2)	25.2 (4.7)	0.228
Home isolation, days	1 (3.8)	0 (2.5)	1 (4)	0.518
Smoking, current	35 (14.3)	9 (12.3)	26 (15.2)	0.356
Smoking, past	29 (11.9)	14 (19.1)	30 (17.4)	0.761
Gender, male	135 (55.3)	38 (52)	97 (56.7)	0.297
**Comorbidities**				
Dementia	70 (28.6)	30 (41)	36 (21)	< 0.001
Diabetes	70 (28.6)	24 (32.8)	46 (26.9)	0.214
Asthma	3 (1.2)	3 (1.7)	0 (0)	0.342
Autoimmune diseases	4 (2)	1 (1.3)	4 (2.3)	0.529
Coronary artery disease	51 (20.9)	21 (28.7)	30 (17.5)	0.038
Myocardial infarction	10 (4)	4 (5.4)	6 (3.5)	0.346
COPD	28 (11.4)	8 (10.9)	20 (11.7)	0.531
Heart failure	44 (18)	20 (27.4)	24 (14)	0.012
Hypertension	155 (63.5)	46 (63)	109 (63.7)	0.513
Malignancy	23 (9.4)	10 (13.7)	13 (7.6)	0.107
Chronic kidney disease	74 (30.3)	40 (54.8)	34 (19.8)	< 0.001
Stroke	11 (4.5)	6 (8.2)	5 (2.9)	0.0801
Cerebrovascular disease	43 (17.6)	19 (26)	24 (14)	0.021
Thyroid disease	12 (4.9)	6 (8.2)	6 (3.5)	0.271
Respiratory failure	12 (4.9)	4 (5.4)	8 (4.6)	0.507
**Clinical presentation**				
Confusional state	58 (23.7)	24 (32.8)	34 (19.8)	0.023
Cough	101 (41.4)	28 (38.3)	73 (42.7)	0.314
Diarrhea	20 (8.2)	5 (6.8)	15 (8.7)	0.414
Fatigue	126 (51.6)	32 (43.8)	94 (54.9)	0.073
Fever	99 (40.5)	30 (41)	69 (40.3)	0.512
Headache	45 (18.4)	9 (12.3)	36 (21)	0.074
Nausea or vomiting	27 (11)	9 (12.3)	18 (10.5)	0.505
Shortness of breath	132 (54)	55 (75.3)	77 (45)	< 0.001
Sore throat	59 (24.1)	19 (26)	40 (23.4)	0.451
Sputum production	25 (10.2)	9 (12.3)	16 (9.3)	0.120
Arthrlagia or myalgia	65 (26.6)	9 (12.3)	56 (32.7)	0.001
OSCI	5.1 (0.8)	5.3 (1.3)	4.9 (0.9)	0.437
**Vital parameters**				
Heart rate	80 (17.5)	80 (23)	80 (14)	0.068
DBP, mmHg	70 (20)	70 (20)	70 (16)	0.355
SBP, mmHg	130 (35)	125 (40)	130 (35)	0.541
MBP, mmHg	90 (21.5)	88 (25)	91 (21)	0.402
Respiratory Rate	18 (6)	24 (7)	18 (4)	< 0.001
Respiratory Rate >20	82 (33.6)	50 (68.5)	32 (18.7)	< 0.001
CGS	15 (2)	13 (4)	15 (0)	< 0.001

*CGS, Coma Glasgow Scale; SBP, Systolic Blood Pressure; DBP, Diastolic Blood Pressure; MBP, Medium Blood Pressure; BMI, Body Mass Index; COPD, Chronic Obstructive Pulmonary Disease; OSCI, Ordinal Scale for Clinical Improvement*.

**Table 2:** Laboratory results in COVID-19 patients at admission.

	**Total**	**Deceased**	**Survivors**	
	**(*n* = 244)**	**(*n* = 73)**	**(*n* = 171)**	***p***
**Laboratory results**				
Albumin, g/dL	3.07 (0.8)	2.7 (0.6)	3.21 (0.6)	< 0.001
Albumin ≤ 2.5	18 (7.7)	15 (21.4)	3 (1.8)	< 0.001
ALT, UI/L	21 (20.5)	23 (24)	20 (17)	0.084
AST, UI/L	26 (20.8)	33 (29)	24 (15)	< 0.001
AST ≥ 42	52 (21.3)	26 (35.6)	26 (15.2)	0.001
CKD-EPI, ml/min	71.9 (50.1)	43.5 (64.5)	79.1 (36.5)	< 0.001
CKD-EPI ≤ 60	94 (38.5)	48 (65.7)	46 (26.9)	< 0.001
CK, U/L	99 (195)	99 (301)	100 (184)	0.177
Creatinin, mg/dL	1 (0.7)	1.3 (1.4)	0.9 (0.5)	< 0.001
Creatinin ≥ 1.2	77 (31.5)	38 (22)	39 (53.4)	< 0.001
D-Dimer, mcg/mL	1 (1.5)	1.86 (3.2)	0.84 (1)	< 0.001
D-Dimer ≥ 4	33 (13.6)	24 (33)	9 (5.2)	< 0.001
LDH, U/L	444.5 (324)	678.5 (596.5)	403 (196.3)	< 0.001
LDH ≥ 450	114 (46.7)	53 (72.6)	61 (35.7)	0.081
NT-PRO-BNP, pg/mL	433.5 (2082.5)	2748.5 (7901)	229.5 (690.3)	< 0.001
NT-PRO-BNP ≥ 125	149 (61)	64 (87.6)	112 (65.4)	< 0.001
Ferritin, ng/mL	621 (678)	785 (773)	487 (676)	0.046
Ferritin ≥ 300	180 (73)	69 (94.5)	117 (68)	0.018
Fibrinogen, mg/dL	527 (253)	562 (274)	515 (216)	0.017
Fibrnogen ≥ 400	192 (78.6)	61 (83.5)	131 (76.3)	0.173
Hb, gr/dL	12.9 (2.8)	12.2 (3.5)	13 (2.4)	0.295
PaO2/FiO2 ratio	295.2 (160.9)	209.2 (213.4)	319 (126)	< 0.001
PaO2/FiO2 ≤ 300	128 (52.4)	54 (73.9)	74 (43.2)	< 0.001
CRP, mg/dL	4.19 (10.2)	10.76 (14)	2.69 (6.2)	< 0.001
CRP ≥ 0.5	198 (81.1)	67 (91.7)	131 (76.6)	< 0.001
PCT, ng/mL	0.11 (0.22)	0.31 (1.13)	0.8 (0.11)	0.002
PCT ≥ 0.5	44 (18)	30 (41)	14 (8)	< 0.001
PLT, cells x 10^4^	20.9 (11.6)	16.6 (16.2)	21.3 (10.8)	< 0.001
PLT ≤ 15 × 10^4^	51 (20.9)	21 (28.7)	30 (17.5)	0.038
Total bilirubin, mg/dL	0.52 (0.5)	0.69 (0.7)	0.51 (0.4)	0.131
Troponin, pg/mL	26.5 (48)	60 (129.5)	18 (23.9)	< 0.001
Troponin ≥ 14	170 (69.6)	66 (90.4)	104 (60.8)	< 0.001
Urea, mg/dL	49 (46)	79.5 (75.5)	40 (28)	< 0.001
Urea ≥ 50	115 (47.3)	55 (75.3)	60 (35)	< 0.001
WBC, cells x 10^3^	7.5 (6.1)	10.5 (7)	6.7 (4.2)	< 0.001
WBC ≤ 4x10^3^	29 (11.9)	4 (5.4)	25 (14.6)	0.030
WBC ≥ 10x10^3^	78 (32)	40 (54.8)	38 (22.2)	< 0.001
Lymphocyte count	1269.5 (880.8)	1,176 (794)	1,300 (883)	0.386

*Data are expressed as median (interquartile range) or number (percentages) accordingly. WBC, white blood cells; PLT, platelet cells; PCT, procalcitonin; Hb, haemoglobin; CRP, C-reactive protein; NT-PRO-BNP, N-terminal pro-brain natriuretic peptide; LDH, lactate dehydrogenase; CK, creatine kinase; CKD-EPI, Chronic Kidney Disease Epidemiology Collaboration; AST, aspartate transaminase; ALT, Alanine transaminase*.

### Comparison of Demographics and Clinical Characteristics Between Survivors and Deceased Patients at Admission

As initial analysis, we sought to compare the profile of patients according to the outcome of interest. Among the demographic features, deceased patients were older, while no differences were observed in gender prevalence or BMI between the groups. Next, we analyzed the differences in the prevalence of comorbidities between survivors and deceased. History of chronic kidney disease, coronary artery disease and heart failure were more frequent in deceased patients, while no differences were found for hypertension or diabetes prevalence. Neurologic features were also significantly different between survivors and deceased: both dementia and cerebrovascular disease prevalence were significantly higher in the deceased groups and, accordingly, new onset confusional state and lower Glasgow Coma Scale were more frequently identified in the group of deceased patients. In addition, while a positive history of COPD or respiratory failure was not more frequent in deceased patients, both a higher respiratory rate and the presence of shortness of breath at admission were common features in deceased patients compared to survivors. On the contrary, the presence of arthralgia or myalgia is a common finding in subjects who survive.

### Comparison of Laboratory Results Between Survivors and Deceased Patients at Admission

Initially, we evaluated the laboratory results at admission of the groups to identify the relevant discriminants of deceased patients. The subjects belonging to each group were then categorized according to the cut-off laboratory reference. In accordance with the comorbidities profile, a higher prevalence of chronic kidney disease, heart failure and coronary artery disease in deceased patients corresponded to a higher troponin T and NT-PRO-BNP levels and also higher urea and creatinine levels with concomitant lower CKD-EPI compared to survivors. In addition, both low platelet count and high white blood cell count and procalcitonin levels were more common in deceased patients compared to survivors with no difference in lymphocytes levels. On the contrary, low white blood cells count was more common in survivors compared to deceased patients. Among the inflammatory markers both CRP and fibrinogen were significantly higher in the deceased group, while no between-group difference was found in ferritin levels. Furthermore, albumin, D-Dimer, LDH and AST levels were significantly higher in deceased patients, while ALT and total bilirubin levels were not different between deceased and survivors.

### Age-Adjusted Univariable Logistic Regression Analysis and Multivariable Model Definition

After identifying the factors that were statistically significant in the deceased group, univariable logistic regression analysis was performed after adjusting for age, as it resulted to be the unique significantly different variable among the demographic characteristics of our study population (Table 3). After adjusting for age, only history of chronic kidney disease remained significant among comorbidities, while age correction did not affect the significance of the factors belonging to clinical presentation and vital parameters. Laboratory results remained all significant apart from NT-PRO-BNP. After ruling out variables from the multivariable analysis according to the differences between-group (Tables 1, 2), we further excluded those variables from the multivariable analysis if they had multicollinearity (urea, creatinine and history of CKD, shortness of breath). Next, we compared the difference between deceased and survivors according to the variables significant in age-adjusted univariable logistic regression by classifying them in binary format according to the laboratory reference range (respiratory rate >20 breaths per minute, albumin ≤ 2.5 g/dL, d-dimer ≥4 mcg/mL, PaO2/FiO2 ratio ≤ 300, AST ≥42 UI/L, CKD-EPI ≤ 60 ml/min, CRP ≥0.5 mg/dL, PLT ≤ 15 × 10^4^, WBC ≥10 × 10^3^, troponin ≥14 pg/mL). Thus, among the 10 variables from the age-adjusted univariable analysis included in the model, the multivariable logistic regression analysis with conditional step-wise backward method retained 7 variables: respiratory rate >20 breaths per minute, albumin ≤ 2.5 g/dL, D-dimer ≥4 mcg/mL, PaO2/FiO2 ratio ≤ 300, CKD-EPI ≤ 60 ml/min, WBC ≥10 × 10^3^, troponin ≥14 pg/mL. Table 4 show the results of the multivariable logistic regression analysis and it reports the 7 variables included in the index.

**Table 3:** Age-adjusted univariable logistic regression analysis for mortality risk.

	**OR (95%CI)**	***p***
Heart failure		0.599
Coronary artery disease		0.363
Chronic kidney disease	2.96 (1.56–5.63)	0.001
Cerebrovascular disease		0.079
Stroke		0.175
Dementia		0.818
Confusional state		0.243
CGS		0.091
Shortness of breath	3.96 (2.03–7.73)	< 0.001
Respiratory Rate > 20 bpm	8 (4.08–15.69)	< 0.001
Albumin <2.5 g/dL	14.72 (3.43–63.25)	< 0.001
AST > 42 U/I	2.62 (1.31–5.25)	0.006
CKD-EPI <60 ml/min	5.22 (2.89–9.41)	< 0.001
Creatinin > 1.2 mg/dL	2.44 (1.29–4.60)	0.006
D-Dimer > 4 mcg/mL	5.57 (2.31–13.40)	< 0.001
NT-PRO-BNP > 125 pg/mL		0.092
PaO2/FiO2 < 300	3.18 (1,65–6,15)	0.001
CRP > 0.5 mg/dL	5.47 (1.79–16.72)	0.003
PLT < 15 × 10^4^ cells	3.5 (1.76–6.94)	0.008
Troponin T > 14 pg/mL	5.15 (1.42–18.62)	0.002
Urea > 50 mg/dL	3.67 (1.9–7.08)	< 0.001
WBC > 10×10^3^ cells	4.39 (2.29–8.42)	< 0.001

*Data are expressed as median (interquartile range) or number (percentages) accordingly. WBC, white blood cells; PLT, platelet cells; PCT, procalcitonin; Hb, hemoglobin; CRP, C-reactive protein; NT-PRO-BNP, N-terminal pro-brain natriuretic peptide; CKD-EPI, Chronic Kidney Disease Epidemiology Collaboration; AST, aspartate transaminase; ALT, Alanine transaminase; CGS, Coma Glasgow scale; bpm: breaths per minute*.

**Table 4:** Multivariable logistic regression analysis for mortality prediction.

	**OR (95% CI)**	***p***
Albumin < 2.5 g/dL	11.577 (1.83–73.12)	0.009
D-dimer > 4 mcg/mL	3.947 (1.12–12.99)	0.024
PaO2/FiO2 < 300	3.437 (1.38–8.56)	0.008
Troponin T > 14 pg/mL	9.851 (2.27–42.76)	0.002
WBC > 10 × 10^3^ cells	2.708 (1.09–6.74)	0.032
Respiratory rate > 20	3.406 (1.43–8.11)	0.006
CKD-EPI < 60 ml/min	2.143 (1.24–5.21)	0.036

*Data are expressed as median (interquartile range) or number (percentages) accordingly. WBC, white blood cells; CKD-EPI, Chronic Kidney Disease Epidemiology Collaboration*.

### Score Identification and Performance Comparison With SOFA, 4C Mortality and Age

**Figure 1:**
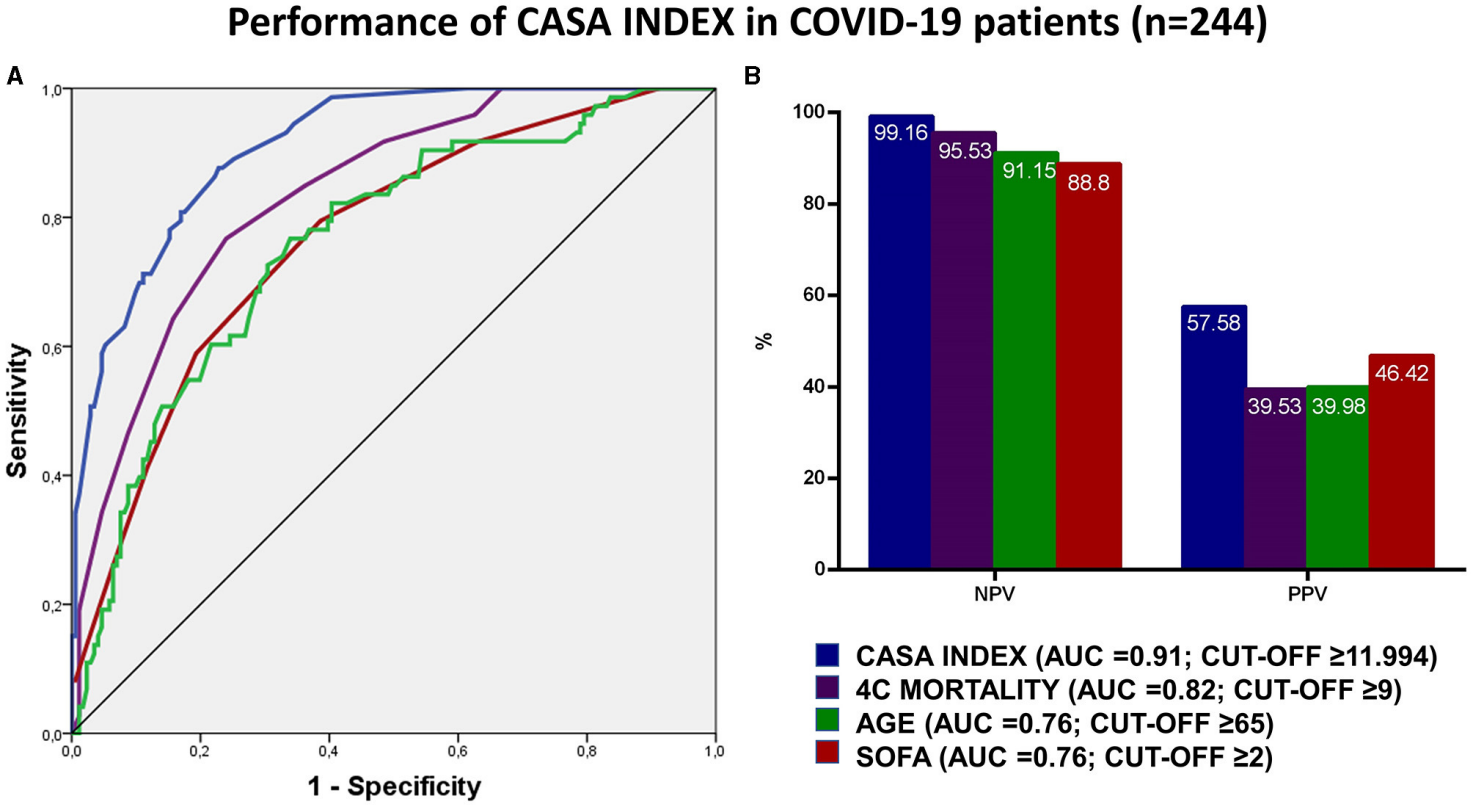
**(A,B)** The figure shows in **(A)** the receiver operating characteristics (ROC) curves for the performance of CASA index, SOFA, 4C mortality and age in predicting mortality in COVID-19 patients at admission. Positive and negative predictive values according to the reference cut-off are reported in **(B)** for the abovementioned scores.

The predictor of mortality score, hereafter CASA (Covid-19 Assessment for Survival at Admission) index, was thus identified by multiplying each variable with the odds ratio derived from the multivariable analysis according to the following formula: Albumin ≤ 2.5 × 11.577 + D-Dimer ≥4 × 3.947 + P/F ≤ 300 × 3.437 + Troponin ≥14 × 9.851 + WBC ≥ 10,000 × 2.708 + Respiratory rate ≥20 × 3.406 + CKD-EPI ≤ 60 ml/min × 2.143. The index showed a high performance with an AUC of 0.91 (95% CI: 0.87 – 0.95), and a sensitivity of 98.6% (95% CI: 92.6 – 99.9), specificity of 69% (95% CI: 61.5 – 75.8) (Supplementary Table 1). The performance of CASA index was significantly superior to each variable analyzed singularly, as compared by AUC. In order to validate the performance of the CASA index, we compared it to the performance of age (AUC = 0.76; 95% CI: 0.69–0.82) and other validated scores, such as SOFA (AUC = 0.76; 95% CI: 0.70–0.83) and 4C mortality (AUC = 0.82; 95% CI: 0.77–0.87) (Figure 1A). After maximizing for sensitivity and specificity we identified that a CASA index score ≥11.994 had a NPV 99.16% (95% CI: 94.4–99.9%) and a PPV 57.58% (95% CI: 52–63%). Next, we compared the NPV and PPV for each score and we observed that the CASA index had the best AUC performance compared to SOFA (*p* < 0.005), age (*p* < 0.0001) and 4c mortality (*p* < 0.0001) (Figure 1B).

## Discussion

After more than 1 year from the onset of the pandemic and the appearance of SARS-COV-2 variants, despite the attempts to start intense vaccination programs, the management of COVID-19 mortality still remains imperfect.

In the balance between rate of admission, mortality risk and disease progression toward severe acute respiratory and multi-organ failure it appears crucial to identify and stratify patients. If highly accurate, such stratification will help to compensate for the uncontrolled virus contagiousness and to prepare highly equipped division for patients needing multi-organ support.

Herein, aiming at stratifying patients for mortality risk at admission, we have developed the seven variables CASA index score in an Italian prospective cohort study involving 244 patients consecutively admitted to the hospital with COVID-19 over 12 months. The CASA index employs only clinical and blood parameters that are commonly available at the time of hospital admission to predict mortality. In addition, the definition of the relevant parameters that increase the risk of mortality has been supported by age-adjustment in our study. While it is widely accepted that older age increases the risk of mortality (25, 26), age alone is not sufficient to stratify patients for intensity of care, while, on the other hand, age remains the main demographic characteristic to consider when building a score for COVID-19.

The identification of patients at risk of mortality is particularly challenging due to the onset of systemic complications of COVID-19 and the evolution toward acute respiratory distress syndrome (ARDS). Traditionally, ARDS presents non-specific manifestations though it should be suspected in those presenting progressive symptoms of respiratory distress, an increasing requirement for oxygen, and alveolar infiltrates on chest imaging. However, several reports suggest that COVID-19 related ARDS is atypical (27–29), mostly due to the discrepancies between blood gas, peripheral saturation (SpO2), lung compliance and the occurrence of systemic complications. Indeed, SpO2 alone might be misleading in assessing COVID-19 pneumonia evolution due to the evidence that these patients present depressed PaO2 values with satisfactory SpO2. This has been defined as “happy” or silent hypoxia, which might lead to an underestimation of the disease severity and influence the clinical decision of the appropriate admission allocation and/or delay intensive treatment (30). Silent hypoxemia is better identified by a significantly increased respiratory rate, which in contrast to hypercapnia that generates dyspnea, seems to not induce dyspnea. This could be due to a possible damage to the afferent hypoxia-sensing neurons, induced by the intense cytokine storm or the direct effect of SARS-CoV-2 (31–34). Compared to lower commonly employed PaO2/FiO2 value ≤ 200 or less, in an attempt to identify more patients in an early phase of disease progression, we included in our index a PaO2/FiO2 value ≤ 300, as supported by the “Berlin definition” of acute respiratory distress (34, 35). Signs of respiratory distress, such as tachypnea, and worsening PaO2/FiO2 were included in our index as they revealed to be independent predictors of mortality. Hence, these factors should be closely monitored, as a sudden and rapidly evolving disease can involve patients in stable conditions (36).

Apart from the lung-specific alterations, several other systemic complications have been reported in severe cases of COVID-19. The interactions between the cardiovascular system, the pulmonary system and kidney function in fatal cases of COVID-19 are complex and multifaceted and also influenced by the activation of the immune system and the coagulation cascade (37). Preexisting coronary artery disease has been frequently observed in fatal cases of COVID-19 and increased troponin levels have been identified as an independent predictor of mortality (38). In COVID-19 the mechanism of troponin elevation is not fully understood, since it might be related to both ischemic injury, less frequently, and non-ischemic damage, such as pneumonia, sepsis, myocarditis, systemic inflammation, pulmonary thrombosis and cardiac adrenergic hyperstimulation during cytokine storm (39). Several reports confirm that all causes of troponin levels elevation are related with the severity of COVID-19 infection and, of note, with disease progression to major complications like multi-organ failure and death (40). In line with previous studies, our results show that normal cardiac troponin levels at admission have a very high negative predictive value for all causes of in-hospital mortality and are a very strong and independent indicators of hospital survival in COVID-19 patients (41). On the other hand, we hypothesize that a high level on admission may help us identify and stratify the subgroup of patients who may progress toward severe complications and death.

To further confirm the complexity of the systemic alterations involving the cardiocirculatory system, several reports suggest that a depressed kidney function at admission, identified by a glomerular filtration rate <60 ml/min, represents an independent predictor of mortality (42), and this also is evidenced by our results. The hematological profile in COVID-19 has been regularly associated with a hypercoagulable status with elevated D-dimer, which in turn might worsen vital organ function (43), and contributes to the development of complications such as ARDS, ICU access, and death (44). The correct identification of COVID-19 subgroups prone to develop either systemic thromboembolism, often associated with a strong immunoinflammatory response (45), or localized forms of coagulopathy, such as the “pulmonary intravascular coagulopathy” (46), remains difficult. Still, the uncontrolled activation of the coagulation system is one of the unique mainstem of COVID-19, leading to increased mortality (29).

In addition, another hematological aspect has emerged in our analysis. While lymphopenia has been invariably linked to the disease itself irrespective of the severity status, leucocytosis is common in patients with aggressive evolution (47), thus adding the disruption of the immune system associated with possible bacterial infection as an additional mortality risk factor, and supporting the relevance of a dynamic monitoring for the early identification of white blood cell increase (48).

Finally, several reports suggest that albumin levels are inversely correlated with the white blood cell count, suggesting that the dysregulated immune response causes an augmented capillary permeability and an extravasation of serum albumin into the interstitial space. Infact, hypoalbuminemia has been already reported as a marker of disease severity and increased hospital mortality in past SARS epidemics (49). It is known that reduced serum albumin levels are found in patients with COVID-19 (50, 51) and in general albumin levels below 2/2.5 g/L are associated with ARDS severity (52). According to other studies on COVID-19 patients, it has been suggested that even a higher serum albumin level cut-off, such as 3.5 g/L at presentation independently increases the risk of death in COVID-19 (49, 53, 54).

Our study has some limitations: firstly, we were unable to evaluate the predictive performance of several existing scores that require a larger number of parameters (for example, APACHE II, PSI), as well as other COVID-19 prognostic scores that use computed tomography findings or uncommonly measured biomarkers. However, we were able to compare the performance of CASA index to the COVID-19 specific 4C mortality index, in addition to age and SOFA. Thus, by including comparisons with pre-existing models, reassurance is provided that equivalent performance cannot be delivered with a simple tool already in use, or at least not one that can be applied on the first hospital day. In addition, treatment regimen, limited by the availability of specific emerging therapy, might have influenced the primary outcome. The sample size involved in the study, while covering a long-term observation, requires validation studies in other cohorts to confirm our findings. Nevertheless, the size of our patient cohort compares favorably to other datasets for model creation. The patient cohort on which the 4C Mortality Score was derived also comprised patients admitted to hospital who were seriously ill (mortality rate of 32.2%) and were of advanced age (median age 72.3 years). Thus, the CASA index is not for use in the community and will likely perform differently in populations at lower risk of death. Further external validation is required to determine whether our index is applicable in other populations.

In conclusion, there is an increased need for tools to stratify patients for intensity of care and to specifically exclude from medium-high intensity divisions those patients not at risk of death. The complex systemic disturbances occurring during COVID-19 remain difficult to ascertain, as does the practical distinction between subjects with a self-limiting disease and those evolving toward acute respiratory syndrome and multi-organ failure. Our index may prove useful to stratify those who are candidates for admission to medium-high intensity of care units and to improve the allocation of equipment and human resources to prepare health system to adapt according to the unpredictable evolution of COVID-19. In addition, the CASA index, due to its easy applicability in emergency departments, facilitates the stratification of medium-low intensity patients to the appropriate division by including a systemic view of the disease, including extrapulmonary manifestations. Covering a wide range of COVID-19 patients requiring non-ICU hospitalization, as expressed by the Ordinal Scale for Clinical Improvement, the CASA index might prove useful in stratifying patients to the appropriate intensity of care and have a large-scale use due to its quick feasibility at admission. Further validation studies are needed to confirm the performance of the index and to test the limits of its generalizability.

## Data Availability Statement

The raw data supporting the conclusions of this article will be made available by the authors, without undue reservation.

## Ethics Statement

The studies involving human participants were reviewed and approved by University of Messina. The patients/participants provided their written informed consent to participate in this study.

## Author Contributions

GB, DLR, CI, MC, AD, MT, NZ, ST, NI, AV, WR, SG, and GS concept and design of the study. CA, FN, VV, GB, CI, MC, NI, and ST data collection, data analysis and interpretation. SG, EV, GS, WR, GB, EI, and AM manuscript preparation. All authors contributed to the article and approved the submitted version.

## Conflict of Interest

The authors declare that the research was conducted in the absence of any commercial or financial relationships that could be construed as a potential conflict of interest.

## Publisher's Note

All claims expressed in this article are solely those of the authors and do not necessarily represent those of their affiliated organizations, or those of the publisher, the editors and the reviewers. Any product that may be evaluated in this article, or claim that may be made by its manufacturer, is not guaranteed or endorsed by the publisher.

## References

[1] Ji W Wang W Zhao X Zai J Li X. Cross-species transmission of the newly identified coronavirus 2019-nCoV. J Med Virol. (2020) 92:433–40. 10.1002/jmv.25682 31967321 PMC7138088

[2] Coronaviridae Study Group of the International Committee on Taxonomy of V. The species Severe acute respiratory syndrome-related coronavirus: classifying 2019-nCoV and naming it SARS-CoV-2. Nat Microbiol. (2020) 5:536–44. 10.1038/s41564-020-0695-z 32123347 PMC7095448

[3] De Larochelambert Q Marc A Antero J Le Bourg E Toussaint JF. Covid-19 mortality: a matter of vulnerability among nations facing limited margins of adaptation. Front Public Health. (2020) 8:604339. 10.3389/fpubh.2020.604339 33330343 PMC7710830

[4] Sesta L Mondello C Cardia L Mondello E Baldino G Spagnolo EV. Covid-19 in italy. Clinical emergency and bioethical perspectives. EuroMediterranean Biomed J. (2020) 15:121–25. 10.3269/1970-5492.2020.15.30

[5] Bradley P Frost F Tharmaratnam K Wootton DG. Research NWCOfR. Utility of established prognostic scores in COVID-19 hospital admissions: multicentre prospective evaluation of CURB-65, NEWS2 and qSOFA. BMJ Open Respir Res. (2020) 7:e000729. 10.1136/bmjresp-2020-000729 33293361 PMC7722817

[6] Gette M Fernandes S Marlinge M Duranjou M Adi W Dambo M. Predict score: a new biological and clinical tool to help predict risk of intensive care transfer for COVID-19 patients. Biomedicines. (2021) 9:566. 10.3390/biomedicines9050566 34070021 PMC8157884

[7] Torres-Macho J Ryan P Valencia J Perez-Butragueno M Jimenez E Fontan-Vela M. The PANDEMYC Score. An easily applicable and interpretable model for predicting mortality associated with COVID-19. J Clin Med. (2020) 9:3066. 10.3390/jcm9103066 32977606 PMC7598151

[8] Lopez-Escobar A Madurga R Castellano JM Velazquez S Suarez Del Villar R Menendez J. Risk score for predicting in-hospital mortality in COVID-19 (RIM Score). Diagnostics (Basel). (2021) 11:596. 10.3390/diagnostics11040596 33810534 PMC8065669

[9] Liu H Chen J Yang Q Lei F Zhang C Qin JJ. Development and validation of a risk score using complete blood count to predict in-hospital mortality in COVID-19 patients. Med (N Y) 2021 2:435–47e4. 10.1016/j.medj.2020.12.013 33521746 PMC7831644

[10] Altschul DJ Unda SR Benton J de la Garza Ramos R Cezayirli P Mehler M. A novel severity score to predict inpatient mortality in COVID-19 patients. Sci Rep. (2020) 10:16726. 10.1038/s41598-020-73962-9 33028914 PMC7542454

[11] Raschke RA Agarwal S Rangan P Heise CW Curry SC. Discriminant accuracy of the SOFA score for determining the probable mortality of patients with COVID-19 pneumonia requiring mechanical ventilation. JAMA. (2021) 325:1469–70. 10.1001/jama.2021.1545 33595630 PMC7890534

[12] Rodriguez-Nava G Yanez-Bello MA Trelles-Garcia DP Chung CW Friedman HJ Hines DW. Performance of the quick COVID-19 severity index and the Brescia-COVID respiratory severity scale in hospitalized patients with COVID-19 in a community hospital setting. Int J Infect Dis. (2021) 102:571–76. 10.1016/j.ijid.2020.11.003 33181332 PMC7833674

[13] Anurag A Preetam M. Validation of PSI/PORT, CURB-65 and SCAP scoring system in COVID-19 pneumonia for prediction of disease severity and 14-day mortality. Clin Respir J. (2021) 15:467–71. 10.1111/crj.13326 33417280

[14] Pokeerbux MR Yelnik CM Faure E Drumez E Bruandet A Labreuche J. National early warning score to predict intensive care unit transfer and mortality in COVID-19 in a French cohort. Int J Clin Pract. (2021) 75:e14121. 10.1111/ijcp.14121 33650136 PMC7995084

[15] Chen Y Zhou X Yan H Huang H Li S Jiang Z. CANPT score: a tool to predict severe COVID-19 on admission. Front Med (Lausanne). (2021) 8:608107. 10.3389/fmed.2021.608107 33681245 PMC7930838

[16] Neto FL Marino LO Torres A Cilloniz C Meirelles Marchini JF Garcia de Alencar JC. Community-acquired pneumonia severity assessment tools in patients hospitalized with COVID-19: a validation and clinical applicability study. Clin Microbiol Infect. (2021) 27:1037.e1–1037.e8. 10.1016/j.cmi.2021.03.002 33813111 PMC8016546

[17] Knight SR Ho A Pius R Buchan I Carson G Drake TM. Risk stratification of patients admitted to hospital with covid-19 using the ISARIC WHO clinical characterisation protocol: development and validation of the 4C Mortality Score. BMJ. (2020) 370:m3339. 10.1136/bmj.m3339 32907855 PMC7116472

[18] Fine MJ Auble TE Yealy DM Hanusa BH Weissfeld LA Singer DE. A prediction rule to identify low-risk patients with community-acquired pneumonia. N Engl J Med. (1997) 336:243–50. 10.1056/NEJM199701233360402 8995086

[19] Coronavirus disease (COVID-2019) R&D. Geneva: World Health Organization. Available online at: http://www.who.int/blueprint/priority-diseases/key-action/novel-coronavirus/en/.

[20] Gallo Marin B Aghagoli G Lavine K Yang L Siff EJ Chiang SS. Predictors of COVID-19 severity: a literature review. Rev Med Virol. (2021) 31:1–10. 10.1002/rmv.2146 32845042 PMC7855377

[21] Ji D Zhang D Xu J Chen Z Yang T Zhao P. Prediction for progression risk in patients with COVID-19 pneumonia: the call score. Clin Infect Dis. (2020) 71:1393–99. 10.1093/cid/ciaa414 32271369 PMC7184473

[22] Gong J Ou J Qiu X Jie Y Chen Y Yuan L. A tool for early prediction of severe coronavirus disease 2019 (COVID-19): a multicenter study using the risk nomogram in Wuhan and Guangdong, China. Clin Infect Dis. (2020) 71:833–40. 10.1093/cid/ciaa443 32296824 PMC7184338

[23] Hsu JC Lee IK Huang WC Chen YC Tsai CY. Clinical characteristics and predictors of mortality in critically Ill influenza adult patients. J Clin Med. (2020) 9:1073. 10.3390/jcm9041073 32283858 PMC7230963

[24] DeLong ER DeLong DM Clarke-Pearson DL. Comparing the areas under two or more correlated receiver operating characteristic curves: a nonparametric approach. Biometrics. (1988) 44:837–45. 10.2307/2531595 3203132

[25] Rapp JL Lieberman-Cribbin W Tuminello S Taioli E. Male sex, severe obesity, older age, and chronic kidney disease are associated with COVID-19 severity and mortality in New York City. Chest. (2021) 159:112–15. 10.1016/j.chest.2020.08.2065 32866462 PMC7455228

[26] Guan WJ Ni ZY Hu Y Liang WH Ou CQ He JX. Clinical characteristics of coronavirus disease 2019 in China. N Engl J Med. (2020) 382:1708–20. 10.1056/NEJMoa2002032 32109013 PMC7092819

[27] Gattinoni L Chiumello D Caironi P Busana M Romitti F Brazzi L. COVID-19 pneumonia: different respiratory treatments for different phenotypes? Intensive Care Med. (2020) 46:1099–102. 10.1007/s00134-020-06033-2 32291463 PMC7154064

[28] Swenson KE Ruoss SJ Swenson ER. The pathophysiology and dangers of silent hypoxemia in COVID-19 lung injury. Ann Am Thorac Soc. (2021) 18:1098–105. 10.1513/AnnalsATS.202011-1376CME 33621159 PMC8328372

[29] Grasselli G Tonetti T Protti A Langer T Girardis M Bellani G. Pathophysiology of COVID-19-associated acute respiratory distress syndrome: a multicentre prospective observational study. Lancet Respir Med. (2020) 8:1201–08. 10.1016/S2213-2600(20)30370-2 32861276 PMC7834127

[30] Tobin MJ Laghi F Jubran A. Why COVID-19 silent hypoxemia is baffling to physicians. Am J Respir Crit Care Med. (2020) 202:356–60. 10.1164/rccm.202006-2157CP 32539537 PMC7397783

[31] U RA Verma K. Happy hypoxemia in COVID-19-a neural hypothesis. ACS Chem Neurosci. (2020) 11:1865–67. 10.1021/acschemneuro.0c00318 32530597

[32] Dhont S Derom E Van Braeckel E Depuydt P Lambrecht BN. The pathophysiology of 'happy' hypoxemia in COVID-19. Respir Res. (2020) 21:198. 10.1186/s12931-020-01462-5 32723327 PMC7385717

[33] Gonzalez-Duarte A Norcliffe-Kaufmann L. Is 'happy hypoxia' in COVID-19 a disorder of autonomic interoception? A hypothesis. Clin Auton Res. (2020) 30:331–33. 10.1007/s10286-020-00715-z 32671502 PMC7362604

[34] Force ADT Ranieri VM Rubenfeld GD Thompson BT Ferguson ND Caldwell E. Acute respiratory distress syndrome: the Berlin definition. JAMA. (2012) 307:2526–33. 10.1001/jama.2012.5669 22797452

[35] Colaneri M Bogliolo L Valsecchi P Sacchi P Zuccaro V Brandolino F. Tocilizumab for treatment of severe COVID-19 patients: preliminary results from SMAtteo COvid19 REgistry (SMACORE). Microorganisms. (2020) 8:695. 10.3390/microorganisms8050695 32397399 PMC7285503

[36] Santus P Radovanovic D Saderi L Marino P Cogliati C De Filippis G. Severity of respiratory failure at admission and in-hospital mortality in patients with COVID-19: a prospective observational multicentre study. BMJ Open. (2020) 10:e043651. 10.1136/bmjopen-2020-043651 33040020 PMC7549463

[37] Gencer S Lacy M Atzler D van der Vorst EPC Doring Y Weber C. Immunoinflammatory, thrombohaemostatic, and cardiovascular mechanisms in COVID-19. Thromb Haemost. (2020) 120:1629–41. 10.1055/s-0040-1718735 33124029 PMC7869061

[38] Gaze DC. Clinical utility of cardiac troponin measurement in COVID-19 infection. Ann Clin Biochem. (2020) 57:202–05. 10.1177/0004563220921888 32255359 PMC7364775

[39] Imazio M Klingel K Kindermann I Brucato A De Rosa FG Adler Y. COVID-19 pandemic and troponin: indirect myocardial injury, myocardial inflammation or myocarditis? Heart. (2020) 106:1127–31. 10.1136/heartjnl-2020-317186 32499236

[40] Metkus TS Guallar E Sokoll L Morrow D Tomaselli G Brower R. Prevalence and prognostic association of circulating troponin in the acute respiratory distress syndrome. Crit Care Med. (2017) 45:1709–17. 10.1097/CCM.0000000000002641 28777195 PMC5600678

[41] Al Abbasi B Torres P Ramos-Tuarez F Dewaswala N Abdallah A Chen K. Cardiac troponin-I and COVID-19: a prognostic tool for in-hospital mortality. Cardiol Res. (2020) 11:398–404. 10.14740/cr1159 33224386 PMC7666590

[42] Trabulus S Karaca C Balkan II Dincer MT Murt A Ozcan SG. Kidney function on admission predicts in-hospital mortality in COVID-19. PLoS ONE. (2020) 15:e0238680. 10.1371/journal.pone.0238680 32881976 PMC7470363

[43] Salabei JK Fishman TJ Asnake ZT Ali A Iyer UG. COVID-19 coagulopathy: current knowledge and guidelines on anticoagulation. Heart Lung. (2021) 50:357–60. 10.1016/j.hrtlng.2021.01.011 33524866 PMC7816593

[44] Arachchillage DRJ Laffan M. Abnormal coagulation parameters are associated with poor prognosis in patients with novel coronavirus pneumonia. J Thromb Haemost. (2020) 18:1233–34. 10.1111/jth.14820 32291954 PMC7262191

[45] Allegra A Di Gioacchino M Tonacci A Musolino C Gangemi S. Immunopathology of SARS-CoV-2 infection: immune cells and mediators, prognostic factors, and immune-therapeutic implications. Int J Mol Sci. (2020) 21:4782. 10.3390/ijms21134782 32640747 PMC7370171

[46] Asakura H Ogawa H. COVID-19-associated coagulopathy and disseminated intravascular coagulation. Int J Hematol. (2021) 113:45–57. 10.1007/s12185-020-03029-y 33161508 PMC7648664

[47] Mehta AA Haridas N Belgundi P Jose WM. A systematic review of clinical and laboratory parameters associated with increased severity among COVID-19 patients. Diabetes Metab Syndr. (2021) 15:535–41. 10.1016/j.dsx.2021.02.020 33711574 PMC7896120

[48] Zhong Z Li H Zhu J Ji P Li B Pang J. Clinical characteristics of 2,459 severe or critically ill COVID-19 patients: a meta-analysis. Medicine (Baltimore). (2021) 100:e23781. 10.1097/MD.0000000000023781 33592834 PMC7870179

[49] Huang J Cheng A Kumar R Fang Y Chen G Zhu Y. Hypoalbuminemia predicts the outcome of COVID-19 independent of age and co-morbidity. J Med Virol. (2020) 92:2152–58. 10.1002/jmv.26003 32406952 PMC7273060

[50] Zhou F Yu T Du R Fan G Liu Y Liu Z. Clinical course and risk factors for mortality of adult inpatients with COVID-19 in Wuhan, China: a retrospective cohort study. Lancet. (2020) 395:1054–62. 10.1016/S0140-6736(20)30566-3 32171076 PMC7270627

[51] Zhang Y Zheng L Liu L Zhao M Xiao J Zhao Q. Liver impairment in COVID-19 patients: A retrospective analysis of 115 cases from a single centre in Wuhan city, China. Liver Int. (2020) 40:2095–103. 10.1111/liv.14455 32239796

[52] Hoeboer SH. Oudemans-van Straaten HM, Groeneveld AB. Albumin rather than C-reactive protein may be valuable in predicting and monitoring the severity and course of acute respiratory distress syndrome in critically ill patients with or at risk for the syndrome after new onset fever. BMC Pulm Med. (2015) 15:22. 10.1186/s12890-015-0015-1 25888398 PMC4381515

[53] Qin C Zhou L Hu Z Zhang S Yang S Tao Y. Dysregulation of immune response in patients with coronavirus 2019 (COVID-19) in Wuhan, China. Clin Infect Dis. (2020) 71:762–68. 10.1093/cid/ciaa248 32161940 PMC7108125

[54] Soeters PB Wolfe RR Shenkin A. Hypoalbuminemia: pathogenesis and clinical significance. JPEN J Parenter Enteral Nutr. (2019) 43:181–93. 10.1002/jpen.1451 30288759 PMC7379941

