# Supplementary material for: The COVID-19 Assessment for Survival at Admission (CASA) Index: A 12 Months Observational Study
Source: Front Med (Lausanne). 2021 Sep 30;8:719976. doi: 10.3389/fmed.2021.719976 (PMC8514624; doi:10.3389/fmed.2021.719976)
Supplement: Supplementary file 1 [file Table_1.DOCX]

**Supplemental Table 1. Performance of predictors of mortality in COVID-19 cohort**

|  | *Cut-off* | *True Positive* | *False Positive* | *False Negative* | *True Negative* | *Sensitivity (%)* | *95% CI* | *Specificity (%)* | *95% CI* |
| --- | --- | --- | --- | --- | --- | --- | --- | --- | --- |
| **CASA** | ≥11.994 | 72 | 53 | 1 | 118 | 98.63 | 92.6 - 99.97 | 69.01 | 61.49 - 75.84 |
| **SOFA** | ≥2 | 61 | 69 | 13 | 101 | 82.43 | 71.83 - 90.3 | 59.41 | 51.63 - 66.86 |
| **AGE** | ≥65 | 66 | 99 | 7 | 72 | 90.41 | 81.24 - 96.06 | 42.11 | 34.61 - 49.88 |
| **4C Mortality** | ≥9 | 70 | 107 | 3 | 64 | 95.89 | 88.46 - 99.14 | 37.43 | 30.16 - 45.14 |

The table shows the sensitivity and specificity with 95% confidence intervals (CI) of each score in predicting mortality for COVID-19 patients (n=244) according to the cut-off.
